# Resection of uterine arteriovenous fistula with temporary occlusion of the internal iliac arteries: Case series and literature review

**DOI:** 10.1097/MD.0000000000039442

**Published:** 2024-08-30

**Authors:** Jianmin Chen, Dong Huang, Jie Yang, Songying Zhang

**Affiliations:** a Assisted Reproduction Unit, Department of Obstetrics and Gynecology, Sir Run Run Shaw Hospital, School of Medicine, Zhejiang University, Zhejiang Key Laboratory of Precise Protection and Promotion of Fertility, Zhejiang Provincial Clinical Research Center for Obstetrics and Gynecology, Hangzhou, China.

**Keywords:** incomplete abortion, retained products of conception, temporary internal iliac artery occlusion, uterine arteriovenous fistula

## Abstract

**Rationale:**

Uterine arteriovenous fistula (UAVF) is a rare vascular abnormality that can cause severe and potentially life-threatening hemorrhage. Uterine artery embolization is a common treatment that may affect ovarian and uterine perfusion and cause fertility problems. We herein report our experience treating 2 patients with UAVF who underwent resection after temporary occlusion of both internal iliac arteries.

**Patient concerns:**

Both patients presented with a large UAVF after incomplete miscarriages in the second trimester. Magnetic resonance imaging revealed a UAVF measuring 3.6 × 2.6 × 2.1 cm over the myometrium of the posterior uterine in case 1, and a UAVF of 7.1 × 4.1 × 4.5 cm was identified in case 2.

**Diagnosis:**

Uterine arteriovenous fistula, retained products of conception.

**Interventions:**

The patients underwent resection of UAVF with temporary occlusion of the internal iliac arteries and hysteroscopic removal of the retained products of conception.

**Outcomes:**

Intraoperative bleeding were minimal. Neither patient exhibited abnormal uterine bleeding at the 6-month follow-up. Follow-up ultrasonography and magnetic resonance imaging showed normal uterine myometrium and endometrium and no residual disease.

**Lessons:**

UAVF resection after temporary occlusion of the internal iliac arteries is a promising treatment approach for UAVF. This technique can reduce intraoperative bleeding and remove the potential hemorrhage-related lesion while preserving fertility.

## 1. Introduction

Uterine arteriovenous fistula (UAVF) is a rare but serious cause of heavy uterine bleeding that can lead to significant morbidity due to abnormal connections between the uterine arterial and venous supply.^[1,2]^ UAVF was first described by Dubreuil and Loubat in 1926.^[3]^ The incidence of UAVF after delivery or abortion is 0.63%.^[4]^ UAVF usually manifests as heavy or irregular vaginal bleeding. A massive sudden gush of vaginal blood is typical after miscarriage, uterine surgery, or cesarean section.^[5]^ Management options include observation, medical treatment, uterine artery embolization (UAE), resection, hysterectomy, or combinations of these.^[6,7]^

Women of reproductive age are primarily affected by UAVF. Therefore, fertility-sparing treatment options are of particular importance. An ideal treatment should eliminate the UAVF lesion, repair the uterine defect, and preserve fertility without massive bleeding. UAE is a first-line treatment for symptomatic UAVF.^[7]^ However, UAE is associated with several potential problems. It may diminish ovarian reserve and reduce uterine perfusion, resulting in a thin endometrium, intrauterine adhesions, amenorrhea, and other complications.^[8,9]^

We implemented a novel surgical approach involving resection of UAVF and RPOC with temporary occlusion of the internal iliac arteries, effectively reducing intraoperative bleeding without affecting the ovarian and uterine blood supply. We herein report our experience with this approach for 2 patients with a large UAVF. This is the first report to describe temporary occlusion of the internal iliac arteries in this context. Both patients provided consent for publication of this report. The institutional review board of our hospital waived the requirement for ethics approval of this case report.

## 2. Case presentation

### 2.1. Case 1

**Figure 1.**
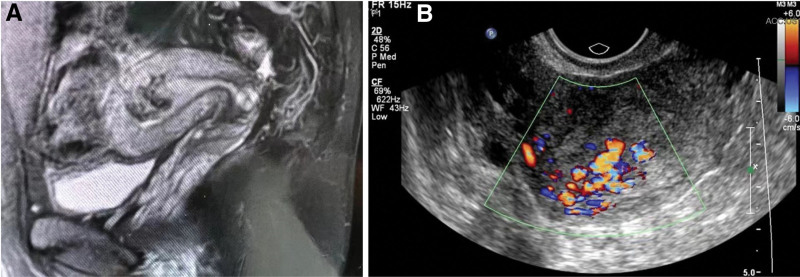
Pelvic MRI (A) and TVS (B) show a UAVF of the right posterior wall measuring 3.6 × 2.6 × 2.1 cm (case 1). MRI = magnetic resonance imaging, TVS = transvaginal ultrasonography, UAVF = uterine arteriovenous fistula.

A 26-year-old woman (gravida 1, para 0) was referred to our department because of irregular vaginal bleeding after dilatation and curettage for inevitable abortion 2 months previously. Her twin embryos had stopped developing at week 12 of gestation. Transvaginal ultrasonography (TVS) with color Doppler showed a UAVF in the myometrium of the posterior uterine wall. The fistula was located close to the endometrium and contained retained products of conception (RPOC). Magnetic resonance imaging (MRI) revealed a 3.6 × 2.6 × 2.1 cm flocculent area of low T2 signal intensity in the uterine cavity and myometrium (Fig. 1). Her human chorionic gonadotropin concentration was 6.53 mIU/mL. UAVF and RPOC were initially diagnosed.

**Figure 2.**
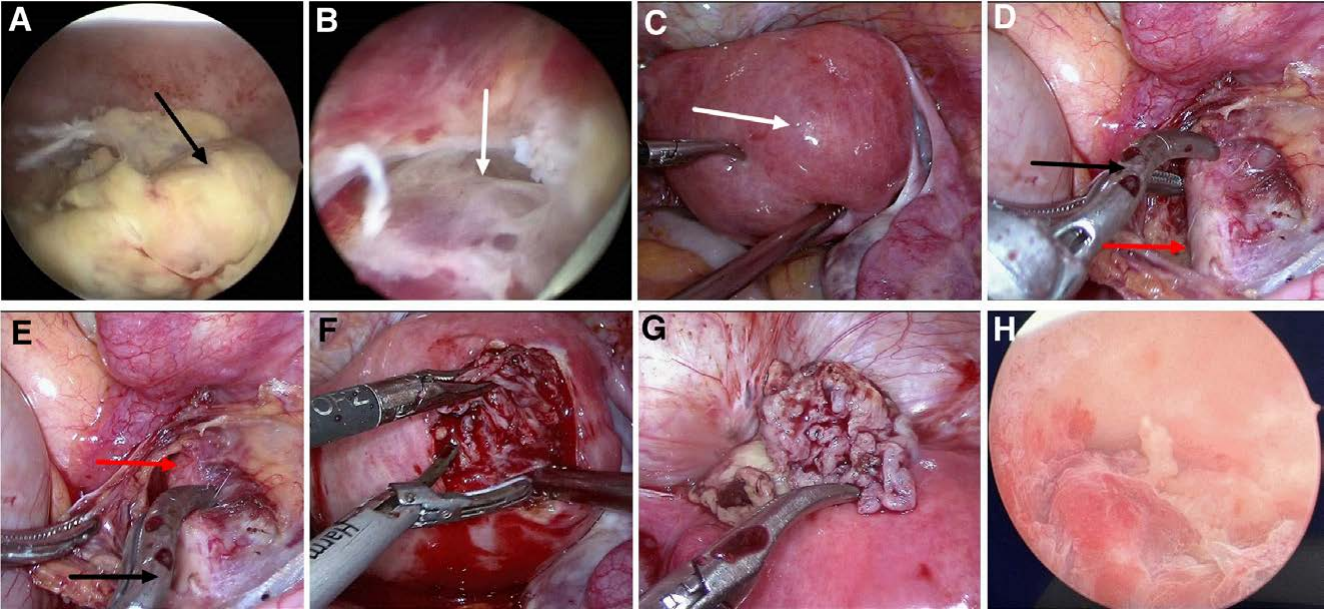
Resection of UAVF with temporary occlusion of the internal iliac arteries (case 1). (A) Hysteroscopy shows RPOC (black arrow) in the uterine cavity. (B) Hysteroscopy reveals a pulsating vascular mass under the RPOC in the right posterior myometrium (white arrow). (C) Laparoscopy shows protrusion of the right posterior wall of the uterus (white arrow). (D, E) The bilateral internal iliac arteries (red arrow) are temporally occluded with a removable curved bulldog clip (black arrow). (F) Scissors are used for sharp dissection of the UAVF lesion, and the uterine incision is sutured in layers. The arterial clips are then removed. (G) The resected UAVF lesion contains thick and tortuous vessels. (H) The organized RPOC is removed under hysteroscopy, and no active bleeding or pulsating vessels are visualized. MRI = magnetic resonance imaging, RPOC = retained products of conception, TVS = transvaginal ultrasonography, UAE = uterine artery embolization, UAVF = uterine arteriovenous fistula.

**Figure 3.**
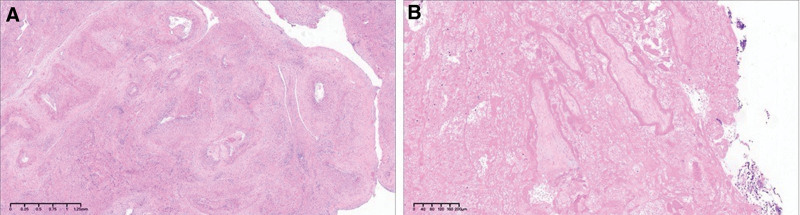
Histopathologic examination revealed UAVF and necrotic villi by HE staining (case 1). (A) Honeycomb-like myometrium and arteriovenous malformation with enlarged lumens and thickened walls. (B) Degenerated villi. UAVF = uterine arteriovenous fistula.

**Figure 4.**
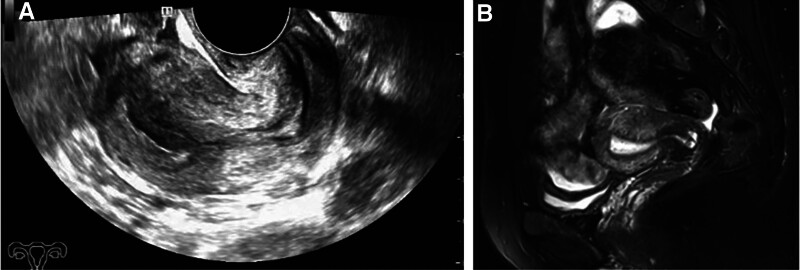
Pelvic TVS (A) and MRI (B) show that the uterus contained normal endometrium and myometrium 3 months after the operation (case 1). MRI = magnetic resonance imaging, TVS = transvaginal ultrasonography.

Laparoscopic surgery was planned for resection of the UAVF lesion after temporary occlusion of the bilateral internal iliac arteries, followed by hysteroscopic resection of the RPOC. The patient was placed in the supine position under general anesthesia. The abdominal cavity was insufflated with carbon dioxide and intra-abdominal pressure was maintained at 15 mm Hg. A 10-mm umbilical trocar was inserted through a supraumbilical port, 3 more ancillary trocars were placed: 1 in the left mid quadrant (5-mm trocar); 1 in the left lower quadrant, 5 cm medial to the left anterior superior iliac crest (5-mm trocar); and 1 at McBurney point (5-mm trocar). A focus of RPOC in the uterine cavity was revealed by Hysteroscopy (Karl Storz, Tuttlingen, Germany; Fig. 2A), equipped with electrosurgical loop. The underlying myometrium contained a thick and tortuous pulsatile vascular mass (Fig. 2B), which was consistent with the imaging findings. Laparoscopy revealed protrusion of the right posterior wall of the uterus, which was softer than the normal surrounding tissues (Fig. 2C). The bilateral internal iliac arteries were dissociated and then temporarily occluded using removable metal clips (bulldog clips; 45mm/4.41N, PL549S, Aesculap, Tuttlingen, Germany; Fig. 2D, E). Pituitrin (6 IU) was diluted with 10 mL of 0.9% saline solution. This was injected into the adjacent myometrium to the UAVF lesion using a long puncture needle connected to a syringe under laparoscopic vision. A monopolar hook was used to incise the bulging myometrium, wherein a widespread tortuous vascular network was visible. An approximately 2.5 × 2.5 cm UAVF lesion was removed (Fig. 2F, G), preserving as much of the endometrium as possible. The uterine incision was sutured continuously in 2 layers with 1# absorbable suture (VCP359H, Ethicon, Angiotech Puerto Rico, Inc), and the metal clips were removed. The organized RPOC were removed under hysteroscopy, with 0.9% saline solution perfusion under 100 mm Hg of pressure. And no active bleeding or obvious pulsating vessels were visualized (Fig. 2H). The amount of saline solution used during hysteroscopy was about 1000 mL. The arterial occlusion time was 30 minutes. The total operative time was 80 minutes with 100 mL of blood loss. After surgery, the patient recovered well and was discharged on postoperative day 2. Histopathologic examination revealed UAVF and necrotic villi (Fig. 3). At the 6-month follow-up, the patient reported no abnormal uterine bleeding, and her menstrual flow was normal. TVS and MRI showed no evidence of UAVF (Fig. 4).

### 2.2. Case 2

A 26-year-old woman (gravida 1, para 0) was admitted because of persistence of RPOC 2 months after dilatation and curettage for inevitable abortion at 20 weeks of pregnancy. She had been pregnant with twins via implantation of 2 embryos. After the dilatation and curettage, she developed a small amount of brown vaginal discharge. Human chorionic gonadotropin was 242 mIU/mL.

**Figure 5.**
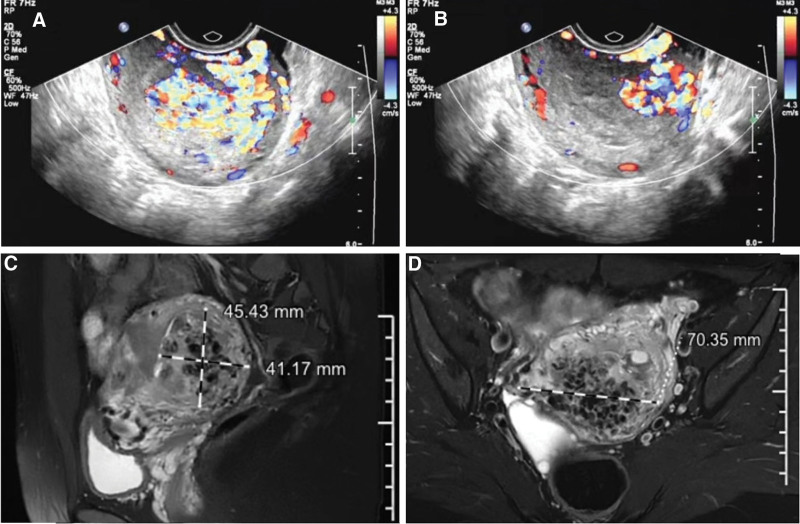
Pelvic TVS (A, B) and MRI (C, D) show a UAVF of the right posterior wall measuring 7.1 × 4.1 × 4.5 cm (case 2). MRI = magnetic resonance imaging, TVS = transvaginal ultrasonography, UAVF = uterine arteriovenous fistula.

After admission, TVS with color Doppler revealed an uneven 3.42- × 1.70-cm echoic mass in the uterine cavity, suggesting RPOC; it was poorly demarcated from the muscular layer of the posterior wall. The myometrium showed sponge-like change and had an abnormally rich blood supply. MRI revealed multiple tortuous flow voids in the right posterior wall of the uterus, which exhibited contrast enhancement (size, 7.1 × 4.1 × 4.5 cm; Fig. 5). The preliminary diagnoses were UAVF and RPOC.

**Figure 6.**
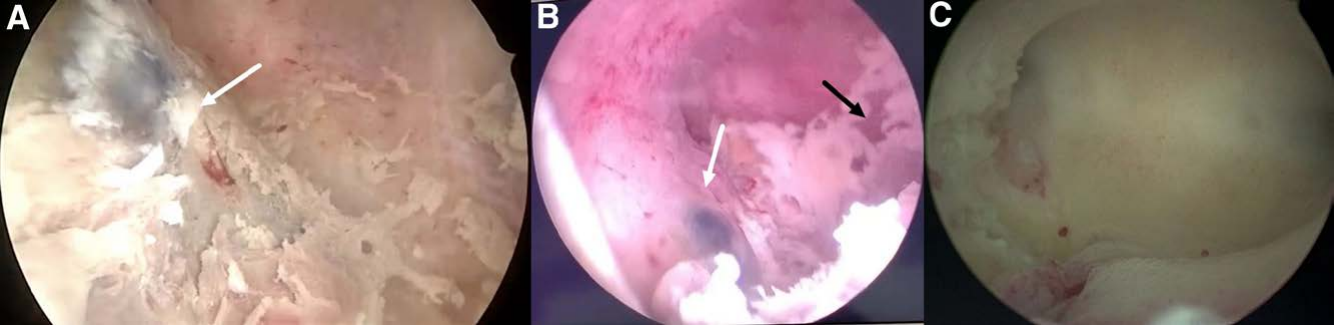
(A, B) Hysteroscopy shows RPOC (black arrow) in the uterine cavity and a pulsating bluish purple protrusion in the right posterior wall (white arrow). (C) Hysteroscopy shows normal uterine cavity without pulsating vascular mass 6 months after the operation (case 2). RPOC = retained products of conception.

**Figure 7.**
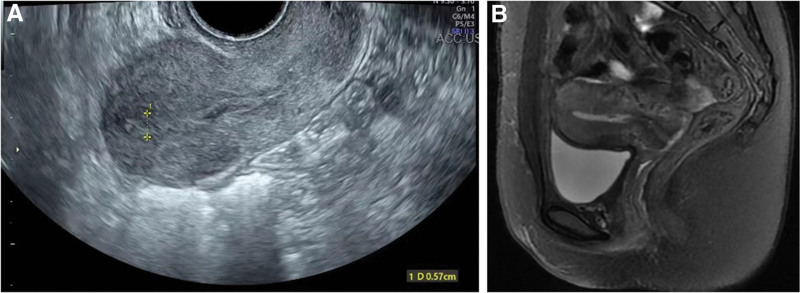
Pelvic TVS (A) and MRI (B) of the uterus show normal endometrium and myometrium 3 months after the operation (case 2). MRI = magnetic resonance imaging, TVS = transvaginal ultrasonography.

The patient underwent laparotomy for resection of the UAVF lesion under temporary occlusion of the bilateral internal iliac arteries and hysteroscopic removal of the RPOC. Hysteroscopy revealed RPOC in the uterine cavity, and a pulsating bluish purple protrusion was seen in the right posterior wall (Fig. 6A, B). Exploratory laparotomy showed that the uterus was enlarged and softened and that the right posterior wall was distended. The bilateral internal iliac arteries were dissociated and then temporarily occluded using removable metal clips as described in case 1. Diluted pituitrin (6 IU) was injected into the adjacent myometrium. An electric knife was used to cut open the right posterior wall of the uterus. The UAVF lesion with honeycomb-like distended arteriovenous vessels was exposed, which involved the entire right posterior wall, extending superiorly to the cervix and inferiorly to the fundus. The serous muscle layer of the posterior uterine wall was dissected to expose the endometrium, an approximately 2.5- × 2.5-cm UAVF lesion was removed, preserving as much of the endometrium as possible. Vessels of the UAVF were sequentially ligated, the uterine incision was sutured in layers. Then the arterial clips were removed. Repeat hysteroscopy showed disappearance of the bluish purple protrusion; the RPOC on the surface of the UAVF were then cleared. The amount of saline solution used during hysteroscopy was about 800 mL. The arterial occlusion time was 35 minutes. The total operative time was 90 minutes with 200 mL of blood loss. The patient was discharged on postoperative day 5 after an uneventful recovery. Histopathologic examination revealed UAVF and necrotic villi. Because the UAVF lesion of this case was very diffuse in the right and posterior wall of the uterus (size, 7.1 × 4.1 × 4.5 cm), therefore 3 injections of a gonadotropin-releasing hormone agonist were administered at 1-month intervals after operation for supplementary treatment. At the 6-month follow-up, the patient reported no abnormal uterine bleeding. Hysteroscopy shows normal uterine cavity without pulsating vascular mass (Fig. 6C). Ultrasonography and MRI showed no evidence of UAVF (Fig. 7).

## 3. Discussion

### 3.1. Pathogenesis and risk factors for UAVF

This case report provides the first description of management of uterine UAVF via temporary occlusion of the internal iliac arteries. UAVF can be congenital or acquired. Acquired UAVF mainly occurs after uterine trauma, such as dilation and curettage (85% of cases), gestational trophoblastic disease, or endometrial carcinoma.^[10]^ Pregnancy-related UAVF is a manifestation of placental bed vessel subinvolution. Placenta accreta, RPOC after induced abortion, and uterine trauma can affect the recovery of the vascular bed and increase the risk of UAVF formation.^[11,12]^ Diseases that can lead to UAVF include subinvolution of the placental vascular bed, gestational trophoblastic disease, placenta accreta in the first trimester, RPOC after induced abortion, and abnormal bleeding after abortion. During early pregnancy, gestational trophoblasts invade the uterine blood vessels and producing new blood vessels to establish contact with mother. Dilatation and curettage, and persistent existence of RPOC lead to incomplete restoration of placental vascular beds, or poor postpartum uterine restoration. Pathological manifestations include abnormal vascularization, where the venous plexus in the uterine muscle layer directly communicates with the uterine artery branch, forming UAVF. Both patients with acquired UAVF described in this report got twin pregnancies after assisted reproduction technology, developed inevitable abortion at mid pregnancy, had irregular vaginal bleeding after dilatation and curettage for a long time, and complicated by RPOC, all these factors affected the recovery of the uterine vascular bed.

### 3.2. UAVF diagnosis

The UAVFs in both of our cases were diagnosed by pelvic TVS and MRI, and hysteroscopy showed pulsating vascular masses. UAVF appears as rich red and blue blood flow signals filling in the anechoic zone on pelvic ultrasonography, showing a mosaic or lake-like change. The arteries are connected to veins and exhibit a mixed arteriovenous spectrum characterized by high speed and low resistance.^[13]^ Computed tomography angiography and MRI can show the structure of UAVFs.^[14]^ Digital subtraction angiography is the gold standard imaging modality for diagnosis.^[15]^ Hysteroscopy can reveal pulsating vascular masses.^[16]^ When UAVF involves the serosa, laparoscopy can also show malformed blood vessels on the surface of the uterus. Histopathologic examination of UAVF demonstrates honeycomb-like myometrium and vessels with enlarged lumens and thickened walls.^[17]^

### 3.3. Treatment of UAVF after temporary occlusion of internal iliac arteries

UAE is the preferred treatment for large lesions with abundant blood flow (peak systolic velocity of > 60–70 cm/s) and patients for whom drug therapy has failed. UAE is also the main treatment for UAVF patients with acute abundant bleeding.^[7]^ However, UAE is associated with potential complications such as post-thrombotic syndrome, injury to ovarian and urinary function, and low uterine perfusion. This can lead to endometrial atrophy, intrauterine adhesions, and even amenorrhea, which increase the risk of miscarriage, preterm birth, fetal growth restriction, postpartum hemorrhage, and placenta accreta.^[8,9,18,19]^ Moreover, UAE can affect the normal blood supply to the pelvic muscles, nerves, and other pelvic organs, potentially resulting in postembolization chronic pelvic pain, lumbosacral pain, and frequent/urgent urination.

Temporary occlusion of the internal iliac arteries before UAVF resection reduces blood flow to the uterus by 48% and lowers the pulse pressure by 85%.^[20]^ This technique is superior to UAE for controlling intraoperative bleeding because the uterine artery has anastomoses with both the vaginal artery and the internal pudendal artery (all of which are branches of the internal iliac artery). To protect ovarian function, efforts should be made to minimize the arterial occlusion time. Previous studies have confirmed uterine artery revascularization 4 months after UAE.^[21]^ In our center, however, arterial occlusion lasts only 30 to 40 minutes, and we have encountered no adverse reactions.

Either laparotomy or laparoscopy can be used to resect a UAVF after temporary occlusion of the internal iliac arteries. If the lesion is localized and protruding toward the uterine cavity, laparoscopic temporary occlusion followed by hysteroscopic local resection can also be considered as an alternative to traditional UAE.

To the best of our knowledge, this is the first report of UAVF resection after temporary occlusion of the internal iliac arteries. This technique eliminates the UAVF focus, repairs the uterine defect, and preserves fertility without massive bleeding. Several previous studies have examined internal iliac artery ligation or temporary occlusion in cesarean scar pregnancy, and no patients developed complications.^[22]^ However, this is the first report of temporary occlusion of the internal iliac arteries in this context. Similarly, 1 report described a case of laparoscopic management of uterine arteriovenous malformation via occlusion of the internal iliac arteries.^[23]^ However, ligation of these arteries can cause irreversible injury, and UAE can affect ovarian and uterine perfusion, leading to various complications. Internal iliac artery temporary blockade with a balloon catheter is also suitable for open surgery such as case 2. Large-scale studies investigating the safety and effectiveness of temporary occlusion of the internal iliac arteries are needed.

In conclusion, resection of UAVF after temporary occlusion of the internal iliac arteries is a promising treatment approach. This technique can reduce intraoperative bleeding and remove the potential hemorrhage-related lesion. Moreover, it preserves fertility without affecting the ovarian function or reducing uterine perfusion, both of which are associated with UAE.

## Acknowledgments

We thank Liwen Bianji (Edanz; https://www.liwenbianji.cn) for editing the English text of a draft of this manuscript.

## Author contributions

**Conceptualization:** Dong Huang, Songying Zhang.

**Data curation:** Jie Yang.

**Writing – original draft:** Jianmin Chen.

**Writing – review & editing:** Jianmin Chen, Songying Zhang.
